# Prevalence and incidence of carbapenem-resistant *K. pneumoniae* colonization: systematic review and meta-analysis

**DOI:** 10.1186/s13643-022-02110-3

**Published:** 2022-11-15

**Authors:** Tewodros Tesfa, Habtamu Mitiku, Mekuria Edae, Nega Assefa

**Affiliations:** 1 Department of Medical Laboratory Sciences, College of Health and Medical Sciences, Haramaya University, P.O.Box 235, Harar, Ethiopia; 2 Hiwot Fana Specialized University Hospital, College of Health and Medical Sciences, Haramaya University, P.O.Box 235, Harar, Ethiopia; 3 School of Nursing Midwifery, College of Health and Medical Sciences, Haramaya University, P.O.Box 235, Harar, Ethiopia

**Keywords:** Colonization, Carbapenem-resistant *K. pneumoniae*, Prevalence, Incidence, systematic review

## Abstract

**Background:**

*Klebsiella pneumoniae* is a gram-negative rod belonging to the order *Enterobacterales* and having a wide distribution in the environment, including the human colon. Recently, the bacterium is one of the known problems in the healthcare setting as it has become resistant to last-resort drugs like carbapenems. The colonized person can serve as a reservoir for his/herself and others, especially in the healthcare setting leading to nosocomial and opportunistic infections. Therefore, we aimed to quantitatively estimate the rate of prevalence and incidence of colonization with carbapenem-resistant *K. pneumoniae*.

**Methods:**

A literature search was conducted on PubMed/MEDLINE, Google Scholar, Science Direct, Cochrane Library, WHO Index Medicus, and university databases. The study includes all published and unpublished papers that addressed the prevalence or incidence of *K. pneumoniae* colonization. Data were extracted onto format in Microsoft Excel and pooled estimates with a 95% confidence interval calculated using Der-Simonian-Laird random-effects model. With the use of *I*^2^ statistics and prediction intervals, the level of heterogeneity was displayed. Egger’s tests and funnel plots of standard error were used to demonstrate the publication bias.

**Results:**

A total of 35 studies were included in the review and 32 records with 37,661 patients for assessment of prevalence, while ten studies with 3643 patients for incidence of colonization. The prevalence of carbapenem-resistant *K. pneumoniae* colonization varies by location and ranges from 0.13 to 22%, with a pooled prevalence of 5.43%. (3.73–7.42). Whereas the incidence of colonization ranges from 2 to 73% with a pooled incidence of 22.3% (CI 12.74–31.87), both prevalence and incidence reports are majorly from developed countries. There was a variation in the distribution of carbapenem resistance genes among colonizing isolates with KPC as a prominent gene reported from many studies and NDM being reported mainly by studies from Asian countries. A univariate meta-regression analysis indicated continent, patient type, study design, and admission ward do not affect the heterogeneity (*p* value>0.05).

**Conclusion:**

The review revealed that colonization with *K. pneumoniae* is higher in a healthcare setting with variable distribution in different localities, and resistance genes for carbapenem drugs also have unstable distribution in different geographic areas.

**Supplementary Information:**

The online version contains supplementary material available at 10.1186/s13643-022-02110-3.

## Background

*Klebsiella pneumoniae* is an omnipresent Gram-negative, non-motile bacterium belonging to the *Enterobacterales* order. It is a common bacterium in the colon and an opportunistic pathogen capable of causing many infections in mammals. Initial colonization is commonly from environmental sources (soil, water, animals, and vegetation) [1]. Despite its dissemination in the environment, the human gut is often a reservoir of *K. pneumoniae* [2]. Although premocolonization precedes *K. pneumoniae* colonization, the exact time of gut colonization is unknown [3].

Based on clinical outcome *K. pneumoniae* strains can be classified to classical and hypervirulent. Nosocomial urinary tract infections, commonly in the elderly and immunocompromised individuals, are frequently caused by the classic *K. pneumoniae* [4, 5]. Hypervirulent *K. pneumoniae* strains, on the other hand, can cause invasive infections with severe complications in immunocompromised and healthy individuals [6].

Carbapenems are commonly used to manage severe infections, especially by *Enterobacterales* having multiple drug resistance [7]. Carbapenems possess broad-spectrum antibacterial activity with a distinctive structure, a carbapenem coupled to a β-lactam ring, confers protection against most β lactamases such as metallo-β-lactamase (MBL) as well as extended-spectrum β-lactamases [8]. Carbapenem resistance is one of the foremost public health concerns as these drugs are the last resort drugs for treating drug-resistant bacteria [9, 10], as in the case of carbapenem-resistant (CR) *Enterobacterales* [11]. Currently, the global health care system is burdened by the high prevalence of carbapenem-resistant *Enterobacterale* (CRE), especially *Klebsiella pneumoniae* and *Escherichia coli* isolates [12–15]. This group of bacteria develops carbapenem resistance mainly by acquiring carbapenemase genes through large transferable plasmids with mobile elements or a combination of β-lactamase overexpansion with weak carbapenem affinity together with reduced permeability or efflux pump [16–18].

Carbapenemases are β-lactamases using carbapenems as hydrolysis substrates, including Ambler classes A, B, and C enzymes. Besides, these strains can also produce ESBLs and/or AmpC enzymes and lose outer membrane porin (OMP) proteins [17].

*Klebsiella pneumoniae* carbapenemase (KPC), Verona integron-encoded metallo-blactamase (VIM), imipenemase metallo-b-lactamase (IMP), and oxacillinase-48 (OXA-48) are among the carbapenemases of global significance [18, 19].

Commonly carbapenem resistance genes occur in bacterial isolates which already have resistance to other multiple drugs [17, 20]. The presence of resistance mechanisms to additional drug classes like all β-lactam drugs and fluoroquinolones exacerbates the problem associated with CRE [20–22]. It has a significant survival advantage for producing organisms as it can target multiple drugs in the environment [23].

Limited treatment options are available to manage infections with CRE, commonly colistin and tigecycline [24]. In some reports, resistance to colistin and tigecycline is indicated [25]. This is a threatening condition as these drugs are of last resort for treating patients infected by multi-drug resistant (MDR) Gram-negative bacteria.

This study aimed to quantitatively estimate the colonization rate of carbapenem-resistant *K. pneumoniae* from fecal samples.

## Methods

### Study protocol

Identification of records, screening of titles and abstracts, and evaluation of full texts for inclusion was performed following the Preferred Reporting Items for Systematic Review and Meta-analysis (PRISMA) flow diagram [26]. Before starting the study, a protocol for the study’s methodology was created, and the PRISMA checklist [27] was closely adhered to throughout this systematic review.

### Identification of records and search strategy

We have set a predetermined search strategy using PICO (Population, Interventions, Comparison, and Outcome). Accordingly, we have looked for all populations with the colonization of carbapenem-resistant *K. pneumoniae*, and intervention and comparison are not applicable as we intend to look for prevalence and incidence.

Literature was searched on electronic databases and indexing services like PubMed/MEDLINE, Science Direct, and other supplementary sources like WHO Index Medicus, Google Scholar, Cochrane library, and university databases. Advanced search, on major databases, was applied to retrieve relevant findings closely related to colonization with carbapenem-resistant *K. pneumoniae.* Carefully selected keywords and indexing terms were used to aid the search in all databases. The selected keywords included “*K. pneumoniae* [MeSH], Carbapenem [MeSH], carrier state [MeSH], Asymptomatic infections [MeSH] “carbapenem-resistant,” carriage, colonization, carbapenemase, “carbapenemase producer”, “carbapenem non-susceptible,” and CRE. Boolean operators (AND, OR), truncation, MeSH, and key terms were used appropriately for the systematic identification of records for the research question. The search was conducted from August 14 to 19, 2021, and all published and unpublished articles available online till the day of data collection were considered. Gray literature from organizations and online university repositories were accessed through Google Scholar with the help of Harzing’s publish or perish 7.0 program [28].

### Screening and eligibility of studies

Identified records were exported to ENDNOTE reference software version 20.0.1 (Thomson Reuters, Stamford, CT, USA) with compatible formats. Records with duplication were traced, documented, and removed with ENDNOTE and manually due to variation in referencing styles across sources. Afterward, the title and abstract of the article were screened by TT, HM, and ME in triplicate with predefined inclusion criteria and full texts evaluation for eligibility.

### Inclusion and exclusion criteria

There were predefined inclusion-exclusion criteria to come up with the final included articles during the initial screening of titles and abstracts and evaluating full texts for eligibility.

Research article which fulfills the following criteria were included and eligible for further analysis

- Article dealing with intestinal colonization of carbapenem-resistant *K. pneumoniae* among patients or community member
- Retrospective and prospective studies addressing the prevalence or incidence of colonization with carbapenem-resistant *K. pneumoniae*
- Online records published in the English language

Articles are excluded for the review if

- It is a review article regardless of the content
- Dealing with infection of *K. pneumoniae* or characterization of isolates from infection site
- If the outcome is incomplete or missing, or if it does not indicate the prevalence or incidence of carbapenem resistant *K. pneumoniae* colonization
- The full article is not accessible after communicating with author (after requesting authors via email and/or Research Gate).

We have utilized the following case definitions for prevalence and incidence of colonization:

- For the incidence of CRKP colonization, a patient who was admitted with a negative CRKP stool culture but later received a positive result at any point after that
- For the prevalence of CRKP colonization, a person who tests positive for stool culture at the time of admission, during admission, or in the community.

### Data extraction

The number of carbapenem-resistant culture-positive results was the outcome of interest. Using a data abstraction format made in Microsoft Excel, significant information about study characteristics (country, first author, year of publication, study design, participant characteristics, study setup, number of culture-positive results (bacterial), type of drug resistance genes, etc.) was extracted by TT and HM (Sheet 1).

### Critical appraisal of studies

Critical appraisal to assess the internal (systematic error) and external (generalizability) validity of studies and to reduce the risk of biases was conducted by TT, HM, and ME separately according to the Joanna Briggs Institute critical appraisal tools for prevalence study, case-control study, and cohort studies [29, 30] and graded.

### Data processing and statistical analysis

Relevant data extracted onto format in Microsoft Excel and exported to STATA 16 (StataCorp LLC, Texas USA) for analyses of outcome measures. DerSimonian and Laird’s random-effects model was applied for the analyses at a 95% confidence level considering variation in true-effect sizes across the population (clinical heterogeneity). Heterogeneity of studies was determined using *I*^2^ statistics and predictive interval. A univariate meta-regression model was performed on study characteristics to assess the possible source of heterogeneity. Egger’s test was used to evaluate the presence of publication bias and presented with funnel plots of the standard error of proportions [31]. A *p* value less than 0.05 (one-tailed) was considered a cutoff point for statistical significance.

### Outcome measurements

The primary outcome measure is the prevalence and incidence of colonization with carbapenem-resistant *K. pneumoniae*.

## Results

### Characteristics of included studies

**Fig. 1:**
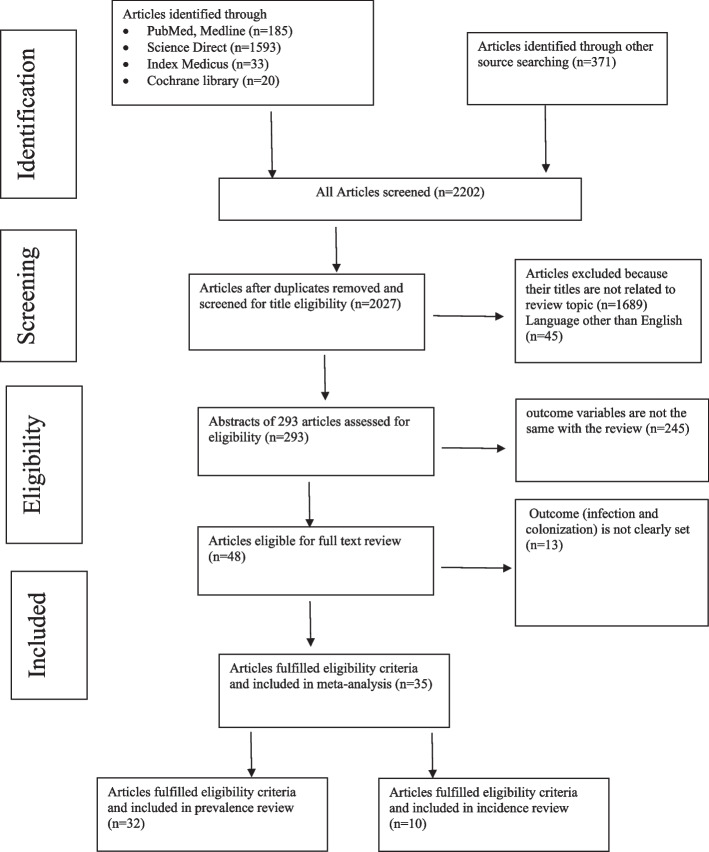
PRISMA flow chart of articles screened for colonization of carbapenem-resistant *K. pneumoniae*

We performed a systematic review following the PRISMA statement [26], and after the initial search of electronic databases and resources, a total of 2202 records were identified from several sources. From these, 175 were duplicate articles occurring in multiple databases and removed with the help of ENDNOTE and manual tracing. The remaining 2027 records were screened based on their titles and abstracts. Totally, 1689 were excluded as the title and abstract are not related to the outcome variable, and 45 due to language. After reviewing the full texts of 293 records for eligibility, 245 articles were also excluded because the outcome of interest was either absent, insufficient, or unclear. Thirty-five articles met the requirements for eligibility and quality assessment (Fig. 1). Of these 32 were used for the analysis of prevalence, and only ten studies for incidence since the studies have reported regarding colonization after admission to a setting.

### Study characteristics

As shown in Table 1, a total of 35 studies with 37,661 participants were included for systematic review. Ten of the included studies were cohort studies [33, 36, 38, 39, 41, 42, 45, 48, 58] and 4 of them case-control [32, 37, 44, 62], while the rest were cross-sectional studies. Two studies [34, 35] have included outpatients while the rest were conducted upon admission and after admission to a setting. Studies were ranked using the Joanna Briggs Institute assessment system, and their average quality scores varied from 5 to 8 (Supplementary file). All studies have applied standard microbiological and molecular techniques. Concerning the country of study, 19 studies were from Asia (8 studies from China and India), ten from Europe, two from Africa, and four from America. Most of the research (more than 50%) were published between 2017 and 2021, with nearly no studies published prior to 2010. Rectal-swabs were used in all investigations to examine the presence of carbapenem-resistant *K. pneumoniae*.

**Table 1:** Characteristics of studies describing the prevalence and incidence of colonization with carbapenem resistant *K. pneumoniae*

Authors	Positive	Sample size	Prevalence	Country	Study design	Type of patient	Ward	Site of sample	Genes
Akturk [32]	85	2805	0.03	Turkey	Case-control	Admitted	NICU & PICU	Rectal swab	
Al Fadhi [33]	7	590	0.01	Kuwait	Cohort	Admitted + community	M/S ICU (adult)	Rectal swab	OXA-181, KPC-2, VIM-1, NDM-5
Antony [34]	1	154	0.01	India	Cross-sectional	Community		Rectal swab	NDM-1
Atterby [35]	2	307	0.01	Cambodia	Cross-sectional	Community	community	Rectal swab	OXA-48
Baraniak [36]	110	17945	0.01	Europe & Israel	Cohort	Admitted	ICU, RU	Rectal swab	KPC-2, KPC-3
Barbadoro [37]	45	2478	0.02	Italy	Case-control	On Admission		Rectal swab	KPC, VIM
Dubby [38]	21	299	0.07	Israel	Cohort	On admission	ICU	Rectal swab	
Errico [39]	58	680	0.09	Italy	Cohort	Admitted	Transplant	Rectal swab	KPC
Ghaith [40]	19	100	0.19	Egypt	Cross-sectional	Admitted	ICU	Rectal swab	NDM-1, VIM, OXA-48,
Giannella [41]	11	237	0.05	Italy	Cohort	On admission	Transplant	Rectal swab	
Giannella [42]	38	553	0.07	Italy	Cohort	On admission	Transplant	Rectal swab	
Girlich [43]	7	77	0.09	Morocco	Cross-sectional	Admitted		Rectal swab	OXA-48
Kang [44]	16	833	0.02	Korea	Case control	On admission	EICU	Rectal swab	KPC
Kiddee [45]	9	275	0.03	Thailand	Cohort	On admission	ICU	Rectal swab	NDM
Kizilates [46]	11	168	0.07	Turkey	Cross-sectional	On admission		Rectal swab	OXA-48, NDM
Liu [47]	42	704	0.06	China	Cross-sectional	On admission	ICU	Rectal swab	KPC, NDM, IMP
Mammina [48]	31	391	0.08	Italy	Cohort	On admission	ICU	Rectal swab	KPC
Maseda [49]	41	254	0.16	Spain	Cross-sectional	On admission	SICU	Rectal swab	OXA-48,
Mohan [50]	9	232	0.04	India	Cross-sectional	Admitted		Fecal sample	NDM-1, VIM
Ohno [51]	2	1487	0.00	Japan	Cross-sectional	Admitted		Fecal sample	
Pan [52]	12	880	0.01	China	Cross-sectional	On admission		Fecal sample	NDM-1, NDM-5, KPC-2, IMP-4
Papadimitriou [53]	40	442	0.09	Greece	Cross-sectional	On admission	ICU	Rectal swab	
Prasa d[54]	46	301	0.15	USA	Cross-sectional	Admitted	LTCF	Rectal swab	KPC-2, KPC-3
Qin [55]	37	243	0.15	China	Cohort	On admission	ICU	Rectal swab	KPC-2
Rios [56]	4	501	0.01	Spain	Cross-sectional	Admitted + outpatient		Rectal swab	KPC-2, OXA-48
Salamao [57]	46	676	0.07	Brazil	Cross-sectional	On admission		Rectal swab	
Salazar-Ospina [58]	5	210	0.02	Colombia	Cohort	Admitted		Rectal swab	
Saseedharan [59]	12	54	0.22	India	Cross-sectional	On admission	ICU	Rectal swab	KPC, NDM, VIM, IMP
Shu [60]	42	202	0.21	China	Cross-sectional	Admitted	ICU	Rectal swab	KPC-2
Tran [61]	260	2233	0.12	Vietnam	Cross-sectional	Admitted	Admitted to hospital	Rectal swab	
Wiener [62]	16	298	0.05	Israel	Case-control	Admitted		Rectal swab	
Xu [63]	25	1052	0.02	China	Cross-sectional	Admitted		Rectal swab	NDM-5, KPC-2, IMP-4

### Prevalence of carbapenem-resistant *K. pneumoniae* colonization

With the exception of two studies from India [34] and Cambodia [35], which are at community surveillance, the majority of the studies, as shown in Sheet 1, were undertaken on admission to hospitals. Patients admitted to ICU (14 studies), transplant recipients [39, 41, 42], and patients admitted to long-term care institutions (one study) [54] have made up the majority of the studies. The highest prevalence was reported from India (12/54, 22%) [59], China (42/202, 21%) [60], Egypt (19/100, 19%) [40], Spain (41/254, 16%) [49], and USA (46/301, 15%) [54], and the lowest from Japan (2/1467, 0.13%) [51].

**Fig. 2:**
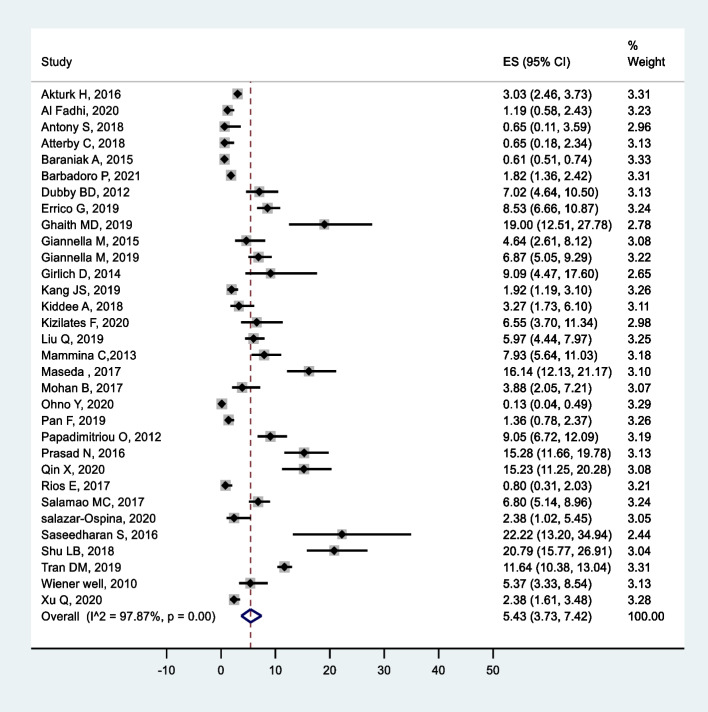
Forest plot for prevalence of Carbapenem-resistant *K. pneumoniae* colonization

**Fig. 3:**
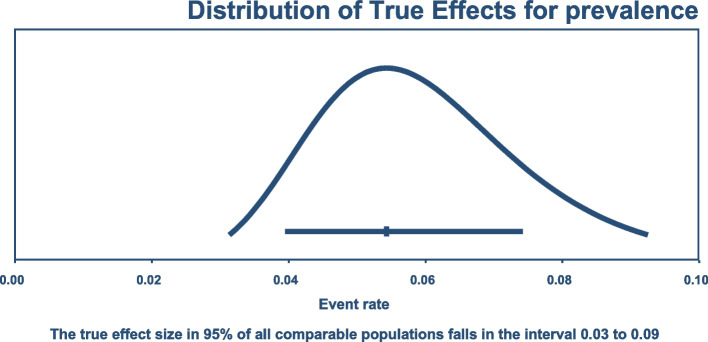
Predictive interval for population prevalence of Carbapenem-resistant *K. pneumoniae*

Average colonization rates ranged from 3.73 to 7.42%, with an *I*^2^ value of 97.87% (Fig. 2). No significant change was observed in the degree of heterogeneity after excluding the known outliers and performing subgroup analysis (Table 2) and sensitivity testing based on the continent, study design, patient type, and admission ward. The predictive interval for the true prevalence of colonization is in the range of 3–9% (Fig. 3).

**Table 2:** Sub-group analysis for prevalence of carbapenem resistant *K. pneumoniae* colonization

Continent	Prevalence	I-squared	Number of studies
Grouping variable			
Asia	4.56 (2.51–7.15)	97.2	14
Africa	14.34 (9.48–19.97)	69.44	2
Europe	6.16 (3.30–9.80)	95.56	9
America	7.39 (2.34–14.84)	92.71	3
**Design**			
Cohort	4.98 (2.07–9.02)	97.77	10
Case-control	2.66 (1.7–3.81)	82.01	4
Cross-sectional	6.52 (3.67–10.09)	97.38	16

The year of study, design, continent, and sample size was not statistically significant in the univariate meta-regression analysis.

### Incidence of carbapenem-resistant K. pneumoniae colonization

Many studies have assessed the incidence of CRKP colonization after admission to ICU except for three studies (two transplant units and one long-term care unit). Five of the published studies are from Asia [33, 38, 44, 45, 64], four from Europe [41, 42, 53, 65], and one from Africa [66]. The highest incidence rate was reported by studies from Greece (164/226, 0.73% and 226/498, 0.45%) and Israel (48/180, 0.27%), and the lowest incidence was from Korea (16/810, 0.02%), Kuwait (22/590, 0.04%), and Thailand (13/206, 0.06%). The length of admission ranges from <8 days to 25 days in published studies.

**Fig. 4:**
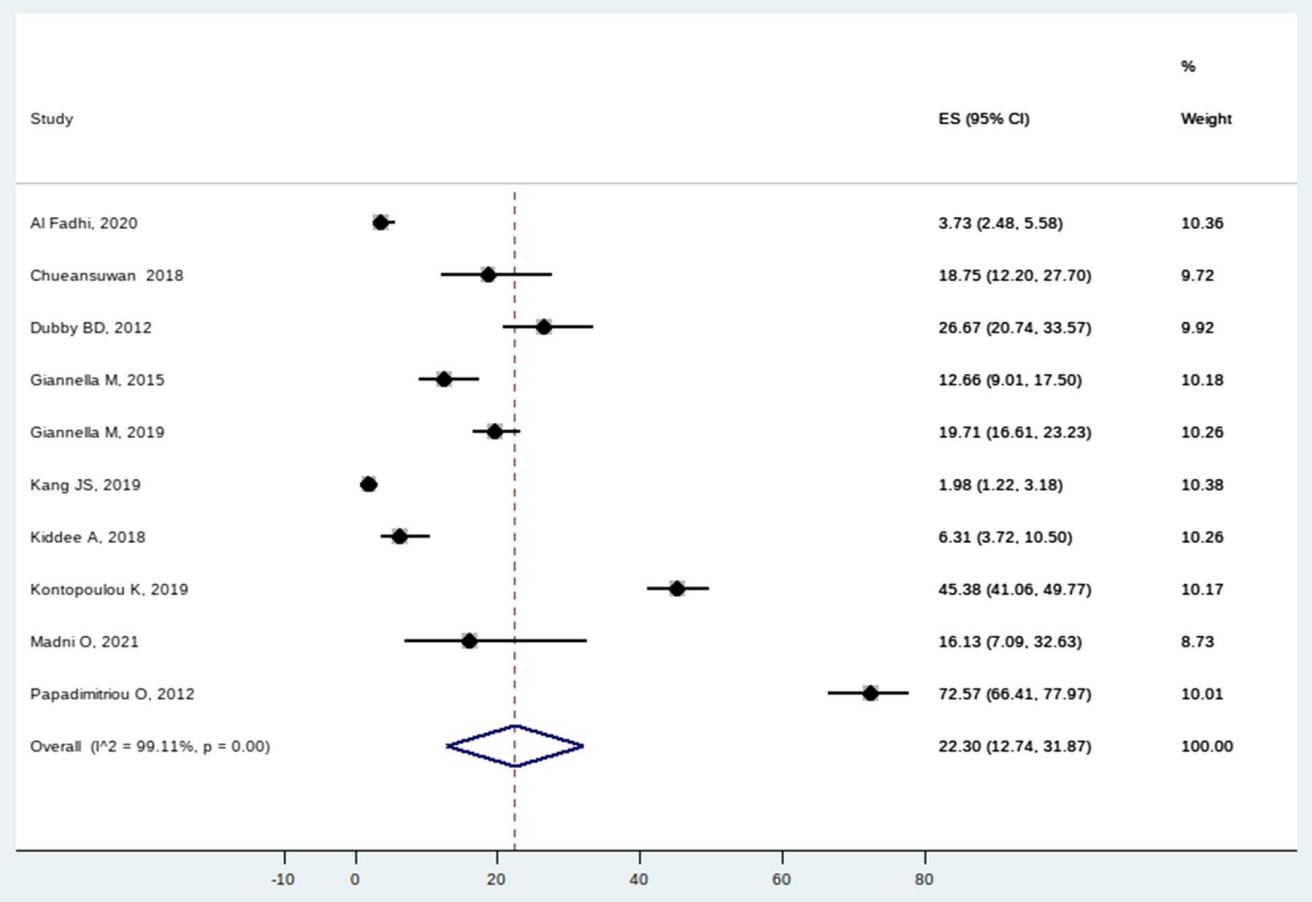
Forest plot for incidence of Carbapenem-resistant *K. pneumoniae* colonization

The incidence of colonization was 22.3% (CI 12.74–31.87) (Fig. 4). There was still high heterogeneity after performing subgroup analysis and sensitivity testing based on the continent, admission ward, and patient type and excluding the known outliers (Table 3).

**Table 3:** Sub-group analysis for Incidence of carbapenem resistant *K. pneumoniae* colonization

Continent	Incidence	I-squared	Number of studies
Grouping variable			
Asia	9.8 (5.17–14.44)	94.77	5
Europe	37.5 (14.18–60.82)	99.15	4
**Design**			
Cohort	25 (23–27.1)	97.69	7
Cross-sectional	59.3 (53–65.2)	98.48	2
**Ward**			
ICU	24.6 (12.37–36.83)	99.36	5
Transplant	17.03 (14.42–19.64)		2

**Fig. 5:**
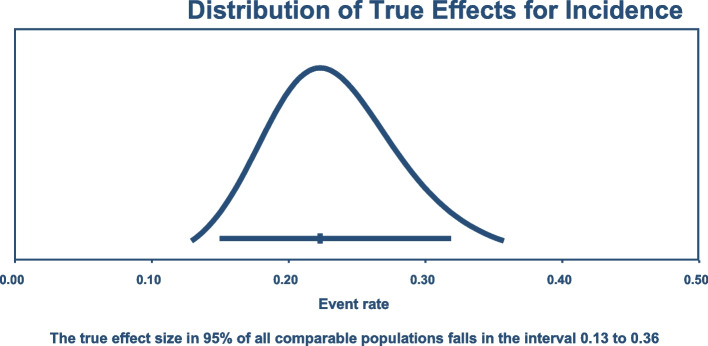
Predictive interval for population incidence of Carbapenem-resistant *K. pneumoniae*

After a univariate meta-regression analysis, the year of study, continent, admission ward, study design, and the sample size were not statistically significant. The 95% predictive value of the true incidence rate of carbapenem-resistant *K. pneumoniae* colonization in the population ranges from 13 to 36% (Fig. 5).

### Drug resistance genes

**Fig. 6:**
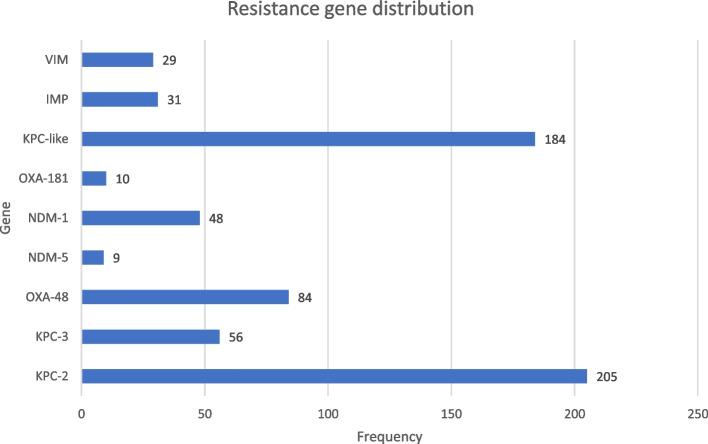
Distribution of carbapenem-resistance genes among colonizing *K. pneumoniae* isolates from different studies

Twenty-two studies have reported the drug resistance genes, including KPC 2, KPC 3, VIM-1, IMP-4, NDM-1, NDM-5, OXA-48, and OXA-181; and KPC was the most commonly reported gene for carbapenemase production (Fig. 6). OXA-48 is the typical variant of OXA, which has been reported in six studies, while OXA-181 is reported only from Kuwait.

The only gene variation class reported from America is KPC, but NDM variants are frequently reported from Asia. Although Africa is represented by two studies, KPC and IMP were not mentioned (Table 4).

**Table 4:** Carbapenamase genes from colonizing *K. pneumoniae* strains in different parts of the world

Continent	Genes
Asia	OXA-181 (10), KPC-2 (98), NDM-5 (9), OXA-48 (13), NDM-1 (23), KPC (48), VIM (14), NDM (12), IMP (17)
Europe	KPC-2 (91), OXA-48 (33), KPC-3 (26), KPC (136), VIM (1)
Africa	OXA-48 (36), NDM-1(25), VIM (14)
America	KPC-2 (16), KPC-3 (30)

### Publication bias

**Fig. 7:**
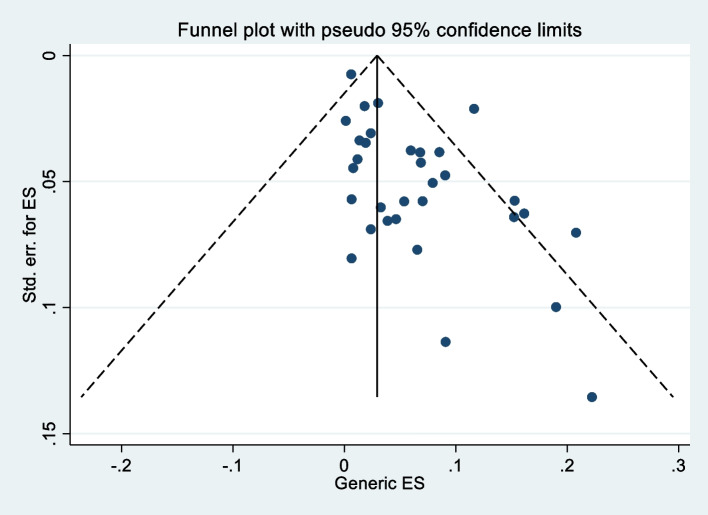
Funnel plot depicting publication bias among studies for prevalence of carbapenem-resistant *K. pneumoniae* colonization

**Fig. 8:**
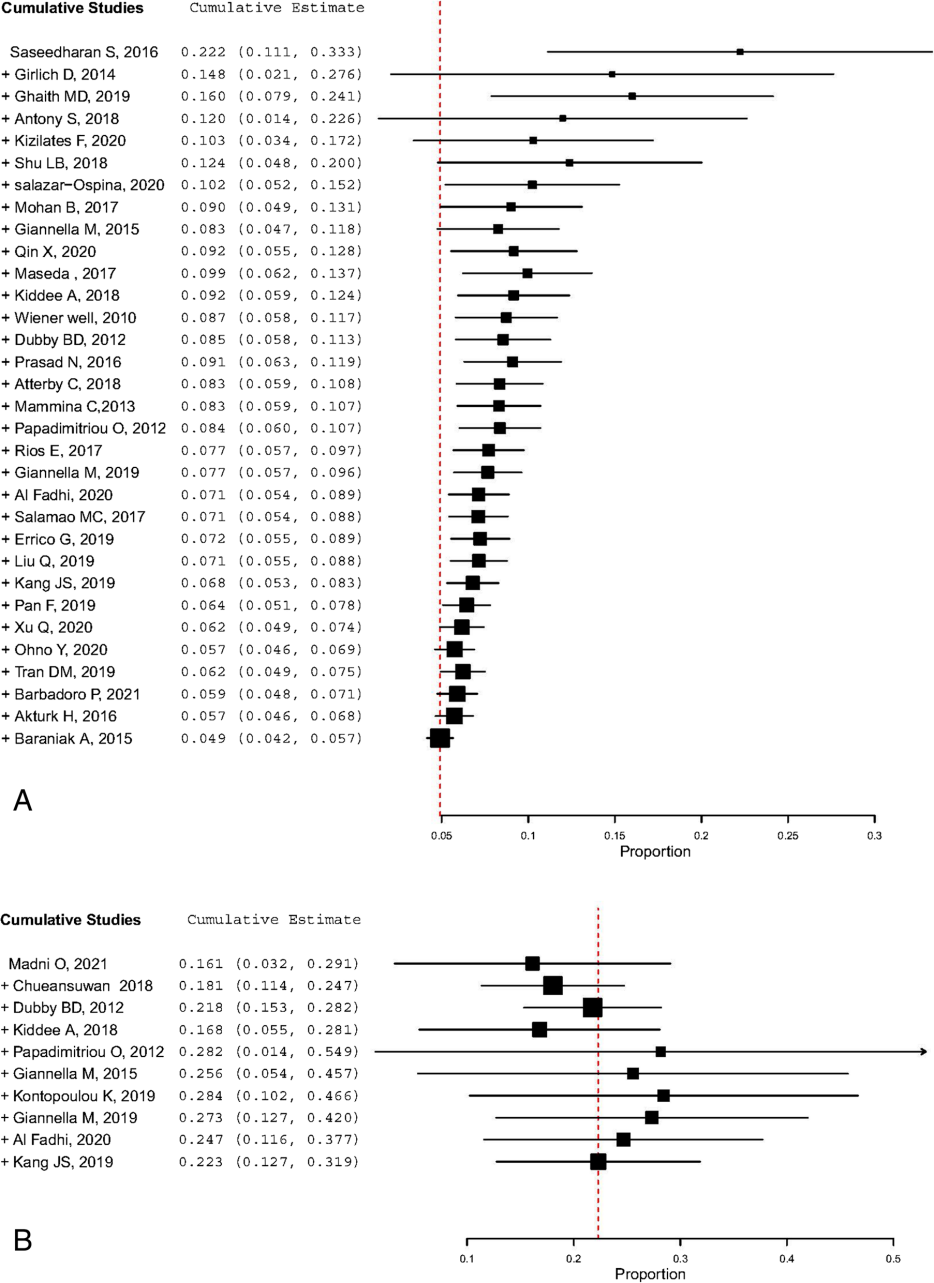
cumulative meta-analysis of studies for prevalence (**A**) and incidence (**B**) of carbapenem resistant K. pneumoniae colonization

Funnel plots of the standard error with proportion supplemented by statistical tests confirmed that there is some evidence of publication bias in studies reporting the prevalence of colonization with carbapenem-resistant *K. pneumoniae* (Egger’s test, *p*= 0.0027) and for incidence of colonization (*p*=0.017) (Fig. 7). In the prevalence of CRKP, a cumulative estimate of value has reduced minimally with an increase sample size, but the precision of the estimate increased. However, in the incidence study, the cumulative estimate with an increased sample size has no directional effect on the incidence estimate (Fig. 8).

## Discussion

This analysis included 35 original studies addressing colonization with carbapenem-resistant *K. pneumoniae*. This is the first report, to the best of our knowledge, on the global frequency of human colonization with carbapenem-resistant *K. pneumoniae*. This report also details the occurrence of resistance genes in different geographic locations. Studies show that colonization with carbapenem-resistant *K. pneumoniae* is increasing from 2010 until now. This review evaluates 37,661 patients from 18 countries between 2010 and 2021.

The pooled prevalence of colonization with carbapenem-resistant *K. pneumoniae* is 5.43%. This finding indicates that a significant portion of the population is colonized with CRKP, which a tremendous burden as colonization could be a risk factor for infection, and one in third of people infected with CRKP will die [67]. The studies show that most of the colonization is from the Asian continent, mainly in China and India, with a frequency of 1.4%. The frequency of carbapenem-resistant *K. pneumoniae* colonization in Europe was 1.2%, 0.3% in the Americas, and 0.07% in Africa. Only two studies have reported colonization with carbapenem-resistant *K. pneumoniae* in Africa, from Egypt and Morocco. This picture may not represent the practical scenario as most countries, especially Africa and America have no data on colonization with CRKP.

Though higher prevalence is reported in China and India, there is still variation within those countries’ reports. In China, the prevalence report ranges from 1 [52] to 21% [60], and also in India, it ranges from 1 [34] to 22% [59]. The pooled prevalence of colonization for the Asian continent is 4.56%. Similarly, reports from Italy show variation in the prevalence rate from 2 to 9% [37, 39], with a 6.16% pooled estimate of prevalence for Europe. This finding indicates that there is no uniform distribution of colonization in different localities, even within a country.

The metaregression analysis on the type of admission ward has not shown any difference in colonization likewise with continents, sample size, and study design. The *I*^2^ statistics showed a high heterogeneity even after subgroup analysis and sensitivity testing, but the prediction interval was relatively narrow (4–7%).

This finding indicates a significant threat posed by the pathogen as colonized peoples will be a source of infection for themselves and others, and people infected with CRKP have a higher healthcare cost and mortality [19, 68].

The incidence of CRKP colonization was reported in 3643 admitted patients, and most of the studies included patients admitted to ICU. As people admitted to ICU are not devoid of several invasive procedures facilitating the introduction of microorganisms to other systems, a high incidence of CRKP colonization poses a higher risk of nosocomial infection with CRKP. CRKP is found commonly on contaminated medical devices and hands of medical personnel in addition to the patient gastrointestinal tract [69]. All patients admitted to ICU virtually have a treatment history of antibiotics, though there are varying reports on the contribution of antibiotics exposure to CRKP colonization [70, 71]. By applying selection pressure to circulating isolates in healthcare facilities, carbapenem use will significantly contribute to the spread of CRKP and increase the risk of colonization for admitted patients [72]. This raises concerns about the efficient application of stewardship and infection control programs with appropriate use of carbapenems in preventing the spread and emergence of CRKP.

European countries have reported a high incidence rate (14.03%), 3.2% in Asian countries, and 0.14% in Africa. For the occurrence of CRKP colonization, only South Africa represents Africa, and Greece and Italy represent Europe. The pooled estimate of colonization incidence is 22.3%, with significant variation between continents (9.8% for Asia and 37.5% for European countries). These continent data variations could be due to data unavailability with the absence of well-organized surveillance programs in healthcare settings of other continents, unlike Europe.

In general, the finding indicates that the risk of colonization is higher in institutional and healthcare settings than in communities where such pathogenic bacteria will have been derived from patients and have continual exposure to last-resort drugs. The reality on the ground could be significantly higher as the data is generated only from a limited number of studies and few countries.

Controlling the spread of drug-resistant bacterial isolates involves strict adherence to the infection control program, early detection, and restricted utilization of antibiotics. Early screening and detection of colonization allow rapid control by contact precautions. Infections with CRKP has important implication due to limited treatment options and the risk of an outbreak from environmental contamination [73, 74]. As the gut is the main reservoir, fecal samples are usually utilized for colonization screening. However, the effectiveness of fecal screening is challenging as these bacteria have a small portion of gut flora coupled with a lack of standardized screening methods [75, 76]. Although the role of carriage screening in various situations has not yet been thoroughly investigated, it may play a crucial role in closely monitoring high-risk groups. In order to reduce the risk of invasive infections, screening can assist set up preventative treatments such as decolonization and decontamination and determine empirical treatment [77]. Such potential interventions must be interpreted in light of a recent review of the interventions to control outbreaks of neonatal healthcare-associated infections, which found that improved swab-based surveillance was ineffective at shortening the number of fatal cases or the length of the episode [78].

Only blaKPC was reported from the Americas, with blaKPC-2 being the most prevalent, while the most frequent carbapenemase-producing gene, blaKPC, was recorded from all continents except Africa. Other genes like blaVIM, blaOXA-like, and blaIMP were reported from different continents. The most reported variant of blaOXA-like was blaOXA-48 in many parts, but one variant, blaOXA-181, was reported only from Kuwait. European countries have not reported blaNDM variants though these variants were common in Asian countries. This finding is in line with [79, 80] where they have indicated that blaNDM-1 was the common variant in India and China. BlaKPC and blaNDM are the most repeatedly reported determinants, and co-occurrence is not a rare finding among clinical isolates [81].

Several earlier studies have demonstrated the widespread occurrence of KPC gene variants, primarily blaKPC-2 and blaKPC-3, as well as NDM and OXA-48 from middle eastern nations [82–85]. Countries in North Africa and the Middle East are thought to be reservoirs for the OXA-48 and NDM-1 [86–90]. The spread of CRKP is becoming polyclonal in Europe from KPC in early reports [91]. Because OXA-48 and IMP are low-level and challenging to trace, their prevalence might be underestimated [43, 92].

Heterogeneity across studies represents a critical limitation in combining observational studies for meta-analysis. We attempted to limit this heterogeneity through the use of relatively narrow inclusion criteria and assessing the quality of included studies via a JBI protocol. For the identified heterogeneity, we use meta-regression to explain potential sources of heterogeneity. Although a large number of patients are part of the studies, the small number of included studies limited the power to assess for publication bias.

The strength of this review lies in its adherence to established methods for conducting systematic reviews, extensive searching, an inclusive date range, and a combined quality assessment of the included studies. Compiling all available evidence on this matter will help Healthcare settings for an informed decision for screening and segregating colonization, organizations working on human health to devise strategies in the decolonization of colonized peoples as well as design a plan to reduce colonization with this resistant pathogen.

## Conclusion and recommendations

In conclusion, this review details the prevalence and incidence of colonization with carbapenem-resistant *K. pneumoniae* and drug resistance genes. Under-developed nations and community people are underrepresented in this study as most studies come from developed countries and medical settings. Studies have reported a variable distribution of colonization with *K. pneumoniae* from different areas ranging from 0.13 to 22% for prevalence in the community or at admission into setup, whereas the incidence colonization ranges from 2 to 73%, with a pooled prevalence of 5.43% and incidence of 22.3%. The incidence rate is relatively higher than the prevalence depicting that colonization is more elevated in healthcare settings than in the community. There was a significant heterogeneity in both prevalence and incidence pooled estimates with I2 statistics, yet, the predictive value for the prevalence is narrow.

On the other hand, various drug resistance genes have been reported from colonizing strains, KPC and NDM being common, in Asian countries mainly. All in all, resistance genes have variable distribution across geography.

As most of the reports are from healthcare settings and in developed countries, there is no clear picture of the problem in the community and the developing world. Thus, we recommend more studies from developing parts of the world and community setting. In general, this review has detailed a higher presence of colonizing *K. pneumoniae* which alarms devising a strategy for decolonization.

## Supplementary Information

**Additional file 1.**

**Additional file 2.**

**Additional file 3.**

## Data Availability

All data generated or analyzed during this study are included in this published article as supplementary information files.

## Abbreviations

CRE: Carbapenemase-producing *Enterbacterales*
CRKP: Carbapenemase-producing *Klebsiella pneumoniae*
ESBL: Extended spectrum β-lactamase
GIT: Gastrointestinal tract
ICU: Intensive care unit
JBI: Joanna Briggs Institute
MBL: Metallo-β-lactamase
OMP: Outer membrane protein
PCR: Polymerase chain reaction
PRISMA: Preferred Reporting Items for Systematic Reviews and Meta-Analyses
USA: United States of America
