# Supplementary material for: Prevalence and incidence of carbapenem-resistant K. pneumoniae colonization: systematic review and meta-analysis
Source: Syst Rev. 2022 Nov 15;11:240. doi: 10.1186/s13643-022-02110-3 (PMC9667607; doi:10.1186/s13643-022-02110-3)
Supplement: Supplementary file 2 — Additional file 2. [file 13643_2022_2110_MOESM2_ESM.docx]

Article appraised for systematic review titled “Prevalence and incidence of carbapenem-resistant *K. pneumoniae* colonization: systematic review and meta-analysis”

| Article author |  | 1 | 2 | 3 | 4 | 5 | 6 | 7 | 8 | 9 | 10 | 11 | Over all |
| --- | --- | --- | --- | --- | --- | --- | --- | --- | --- | --- | --- | --- | --- |
| Al Fadhi, 2020 | cohort | Y | Y | Y | U | U | Y | Y | Y | Y | U | Y |  |
| Dubby BD, 2012 | cohort | Y | Y | Y | U | U | Y | Y | Y | Y | NA | Y |  |
| Errico G, 2019 | cohort | Y | Y | Y | U | U | Y | Y | Y | Y | NA | Y |  |
| Giannella M, 2015 | cohort | Y | Y | Y | Y | Y | Y | Y | Y | Y | Y | Y |  |
| Giannella M, 2015 | cohort | Y | Y | Y | Y | Y | Y | Y | Y | Y | Y | Y |  |
| Kiddee A, 2018 | cohort | Y | Y | Y | U | U | Y | Y | Y | Y | NA | Y |  |
| Mammina C,2013 | Cohort | Y | Y | Y | U | U | Y | Y | Y | Y | Y | Y |  |
| Qin X, 2020 | cohort | Y | Y | Y | N | N | Y | Y | Y | Y | U | Y |  |
| salazar-Ospina, 2020 | cohort | Y | Y | Y | U | U | Y | Y | Y | Y | U | Y |  |

Reviewer _Tewodros Tesfa ___ Date_______________________

Article appraised for systematic review titled “Prevalence and incidence of carbapenem resistant *K. pneumoniae* colonization: systematic review and meta-analysis”

| Article author |  | 1 | 2 | 3 | 4 | 5 | 6 | 7 | 8 | 9 | 10 | Over all |
| --- | --- | --- | --- | --- | --- | --- | --- | --- | --- | --- | --- | --- |
| Akturk H, 2016 | case-control | U | N | Y | Y | Y | U | U | Y | Y | Y |  |
| Barbadoro P, 2021 | Case Control | Y | Y | Y | Y | Y | U | U | Y | Y | Y |  |
| Kang JS, 2019 | case control | U | U | Y | Y | Y | N | N | Y | Y | Y |  |
| Wiener well, 2010 | Case-control | Y | N | Y | Y | Y | U | U | Y | Y | Y |  |

Reviewer ____Tewodros Tesfa___ Date_______________________

Article appraised for systematic review titled “Prevalence and incidence of carbapenem resistant *K. pneumoniae* colonization: systematic review and meta-analysis”

| Article author |  | 1 | 2 | 3 | 4 | 5 | 6 | 7 | 8 | 9 | Over all |
| --- | --- | --- | --- | --- | --- | --- | --- | --- | --- | --- | --- |
| Akturk H, 2016 | Case-control |  |  |  |  |  |  |  |  |  |  |
| Al Fadhi, 2020 | Cohort |  |  |  |  |  |  |  |  |  |  |
| Antony S, 2018 | Cross-sectional | Y | U | Y | Y | Y | Y | Y | Y | Y |  |
| Atterby C, 2018 | Cross-sectional | Y | Y | U | Y | Y | Y | Y | Y | Y |  |
| Baraniak A, 2015 | Cross- sectional | Y | Y | Y | N | Y | Y | Y | Y | Y |  |
| Barbadoro P, 2021 | Case control |  |  |  |  |  |  |  |  |  |  |
| Dubby BD, 2012 | Cohort |  |  |  |  |  |  |  |  |  |  |
| Tran DM, 2019 | Cross-sectional | Y | Y | Y | Y | Y | Y | Y | Y | Y |  |
| Errico G, 2019 | Cohort |  |  |  |  |  |  |  |  |  |  |
| Ghaith MD, 2019 | Cross-sectional | Y | U | Y | N | Y | Y | Y | Y | Y |  |
| Giannella M, 2015 | Cohort |  |  |  |  |  |  |  |  |  |  |
| Giannella M, 2015 | Cohort |  |  |  |  |  |  |  |  |  |  |
| Girlich D, 2014 | Cross-sectional | Y | U | N | N | Y | Y | Y | Y | Y |  |
| Kang JS, 2019 | Case control |  |  |  |  |  |  |  |  |  |  |
| Kiddee A, 2018 | Cohort |  |  |  |  |  |  |  |  |  |  |
| Kizilates F, 2020 | Cross-sectional | Y | Y | U | Y | Y | Y | Y | Y | Y |  |
| Liu Q, 2019 | Cross-sectional | Y | U | Y | N | Y | Y | Y | y | y |  |
| Mammina C,2013 | Cohort |  |  |  |  |  |  |  |  |  |  |
| Maseda , 2016 | Cross-sectional | Y | U | Y | Y | Y | Y | Y | Y | Y |  |
| Mohan B, 2017 | Cross-sectional | Y | Y | Y | N | Y | Y | Y | Y | Y |  |
| Ohno Y, 2020 | Cross-sectional | Y | Y | Y | N | Y | Y | Y | N | Y |  |
| Pan F, 2019 | Cross-sectional | Y | Y | Y | N | Y | Y | Y | N | Y |  |
| Prasad N, 2016 | Cross-sectional | Y | U | Y | N | Y | Y | Y | Y | Y |  |
| Qin X, 2020 | Cohort |  |  |  |  |  |  |  |  |  |  |
| Rios E, 2017 | Cross-sectional | Y | U | U | N | Y | Y | Y | N | Y |  |
| Salazar-Ospina, 2020 | Cohort |  |  |  |  |  |  |  |  |  |  |
| Salamao MC, 2017 | Cross-sectional | Y | Y | Y | N | Y | Y | Y | Y | Y |  |
| Saseedharan S, 2016 | Cross-sectional | Y | Y | N | N | Y | Y | Y | Y | Y |  |
| Shu LB, 2018 | Cross-sectional | Y | Y | U | N | U | Y | Y | U | Y |  |
| Wiener well, 2010 | Cross-sectional |  |  |  |  |  |  |  |  |  |  |
| Xu Q, 2020 | Cross-sectional | Y | Y | Y | N | Y | Y | Y | N | Y |  |
| Papadimitriou MO, 2011 | Cross-sectional | Y | Y | U | Y | Y | Y | Y | U | Y |  |

Reviewer ___Tewodros Tesfa__ Date_______________________
