# Supplementary material for: Prevalence and incidence of carbapenem-resistant K. pneumoniae colonization: systematic review and meta-analysis
Source: Syst Rev. 2022 Nov 15;11:240. doi: 10.1186/s13643-022-02110-3 (PMC9667607; doi:10.1186/s13643-022-02110-3)
Supplement: Supplementary file 3 — Additional file 3. [file 13643_2022_2110_MOESM3_ESM.pdf]

Prevalence and Incidence of Carbapenem Resistant *K. pneumoniae* Colonization: systematic review and Meta-analysis protocol

Author's Affiliations

Tewodros Tesfa, Department of Medical Laboratory Sciences, College of Health and Medical Sciences, Haramaya University, P.O.Box 235, Harar, Ethiopia, tewodrost1@gmail.com

Habtamu Mitiku, Department of Medical Laboratory Sciences, College of Health and Medical Sciences, Haramaya University, P.O.Box 235, Harar, Ethiopia, habtemit@gmail.com

Mekuria Edea, Hiwot Fana Specialized University Hospital, College of Health and Medical Sciences, Haramaya University, P.O.Box 235, Harar, Ethiopia, meku.grace@gmail.com

Nega Assefa, School of Nursing Midwifery, College of Health and Medical Sciences, Haramaya University, P.O.Box 235, Harar, Ethiopia, negaassefa@yahoo.com

Corresponding author: Tewodros Tesfa, tewodrost1@gmail.com

Review Question

The aim of this review is to assess the best available evidence regarding the prevalence and incidence of carbapenem resistant K. pneumoniae and the major drug resistance genes

The objectives are:

- To determine the prevalence of carbapenem resistant *K. pneumoniae* colonization from community
- To determine the incidence of carbapenem resistant *K. pneumoniae* colonization in healthcare setting
- To assess the major drug resistance genes

Search Strategy

The search strategy aims to find both published and unpublished studies. We will search the following electronic databases: MEDLINE, EMBASE, the Cochrane Library, Hinari, WHO Global Health database, Scopus and Google Scholar. A three-step search strategy will be utilized in this review. An initial limited search of the electronic databases will be undertaken followed by an analysis of the text words contained in the title and abstract, and of the index terms used to describe the article. A second search using all identified keywords and index terms will then be undertaken across all included databases. Thirdly, the reference list of all identified reports and articles will be searched for additional studies. Subject headings relevant to each database will also be used, for example, MeSH for MEDLINE. In addition, google and hand searching will be employed to retrieve grey literature. Initial keywords to be used will be: *K. pneumoniae*, Carbapenem, carrier state, Asymptomatic infections, ‘'carbapenem-resistant", carriage, colonization, carbapenemase, "carbapenemase producer”, “carbapenem non-susceptible", and CRE and entry terms for K. pneumoniae, carbapenem resistant, carriage and colonization. Retrievals will be limited to English Language.

Inclusion and Exclusion criteria

For this review community members or patients within a healthcare setting with intestinal colonization of carbapenem-resistant *K. pneumoniae* are the study participants. Both retrospective and prospective studies addressing the prevalence or incidence of colonization with carbapenem-resistant *K. pneumoniae* will be included regardless of the participants/ patient clinical characteristics. Regarding the language of publication, online records published in the English language will be considered for further eligibility assessment.

Review and original articles dealing with infection of *K. pneumoniae* will be excluded during the initial screening. Records with missing or incomplete outcomes, unrelated outcome measures, and inaccessible full texts (after requesting authors via email and/or Research Gate) will be excluded.

Case definitions for prevalence and incidence of colonization with carbapenem resistant *K. pneumoniae* are as follows

- For the incidence of CRKP colonization: a patient who is negative for CRKP stool culture at admission and have a culture-positive result at any time after admission within a setting
- For the prevalence of CRKP colonization: A patient who is positive for stool culture before admission to setup, at admission, or community
- Healthcare setting: Health care setting in this article follows the CDC definition as broad array of services and places where healthcare occurs, including acute care hospitals, urgent care centers, rehabilitation centers, nursing homes and other long-term care facilities, specialized outpatient services (e.g., hemodialysis, dentistry, podiatry, chemotherapy, endoscopy, and pain management clinics), and outpatient surgery centers.

Condition to be studied

Colonization with carbapenem resistant *K. pneumoniae* is carriage of this potentially pathogenic bacterium in the intestinal tract after exposure to environmental source.

Population

- The study subjects should have fecal sample or rectal swab sample for microbiological analysis
- People of all age range, gender, ethnic background
- Should not have infection with *K. pneumoniae* at the time of screening

Intervention(s), exposure(s)

Not applicable: since we will estimate the prevalence, we will not have intervention group

Comparator(s)/control

Not applicable: this systematic review will estimate the pooled prevalence or incidence so we don't have control group.

Context

The review will consider studies conducted at community level, during hospital admission or after hospital admission regardless of the geographic location.

Main outcome(s)

The magnitude of colonization with carbapenem resistant *K. pneumoniae* within the community as well as after admission to the healthcare setting.

Additional outcome(s)

The major drug resistance genes from carbapenem resistant *K. pneumoniae* isolates

Assessment of methodological quality

Retrieved full articles will be assessed by three independent reviewers for methodological validity

before inclusion in the review using standardized critical appraisal instruments from the Joanna Briggs Institute Meta-Analysis of Statistics Assessment and Review Instrument. Any disagreements that arise between the reviewers will be resolved through discussion, or with a fourth reviewer.

Data extraction

Data will be extracted using a data extraction tool that is prepared on Microsoft Excel. Information about primary author, study setting, study design, type of participant, region, year of study, year of publication, number of study participants, number of cases, carbapenem resistant isolates and drug resistance genes will be extracted from each included study.

Strategy for data synthesis

A STATA version 16 statistical software will be used for meta-analysis. A quantitative synthesis will be used if the included studies are sufficiently homogenous. Publication bias will be assessed by visual inspection of the funnel plots, and supplemented with a formal statistical testing using the Egger/Begger's tests.

Analysis of subgroups or subsets

Based on the test statistics if there is a significant heterogeneity among studies (I^2 greater than 75%). Subgroup analysis will be conducted by continent, study design, patient type, and admission ward.

Organizational affiliation of the review

Haramaya University

Funding sources/sponsors

None

Anticipated duration of study

June 2021- December 2021
